# An Emerging Trend of At-Home Uroflowmetry—Designing a New Vibration-Based Uroflowmeter with Artificial Intelligence Pattern Recognition of Uroflow Curves and Comparing with Other Technologies

**DOI:** 10.3390/diagnostics15141832

**Published:** 2025-07-21

**Authors:** Vincent F. S. Tsai, Yao-Chou Tsai, Stephen S. D. Yang, Ming-Wei Li, Yuan-Hung Pong, Yu-Ting Tsai

**Affiliations:** 1 Department of Urology, Taipei Tzu-Chi Hospital, Buddhist Tzu-Chi Medical Foundation, New Taipei City 231, Taiwan; ntubala@gmail.com (V.F.S.T.); beastailmw@gmail.com (M.-W.L.); 2 Department of Urology, National Taiwan University Hospital, Taipei 100, Taiwan; 3 Department of Urology, Ten-Chen General Hospital, Taoyuan 326, Taiwan; 4 Bachelor’s Program of Precision System Design, Feng Chia University, Taichung 407, Taiwan; 5 Master’s Program of Electro-Acoustics, Feng Chia University, Taichung 407, Taiwan

**Keywords:** urination disorders, urodynamics, vibration, artificial intelligence

## Abstract

**Background/Objectives**: For aging men experiencing lower urinary tract symptoms (LUTS), bladder diaries (BD) and uroflowmetry (UFM) are commonly used non-invasive diagnostic tools. However, bladder diaries often suffer from subjectivity and incomplete data, while traditional hospital-based uroflowmetry lacks convenience and repeatability. Therefore, there is a growing need for a user-friendly, artificial intelligence (AI)-powered at-home uroflow monitoring solution. This study aims to develop a novel, vibration-based home uroflowmetry system capable of recognizing uroflow curve patterns and measuring voiding parameters, and to compare its performance with other existing home-based uroflowmetry methods. **Methods**: Seventy-six male participants, all of whom provided informed consent, underwent uroflowmetry to assess voiding symptoms. An accelerometer affixed to the uroflowmeter’s urine container captured vibration signals, which were used to calculate the root mean square (RMS) values and maximum amplitude (Mmax). Simultaneously, the uroflowmeter recorded standard voiding parameters and generated uroflow curves. These vibration signals were then analyzed using a convolutional neural network (CNN) to classify six distinct uroflow curve patterns, aiding in diagnostic evaluation. **Results**: Seventy-six participants’ voiding volume ranged from 50 mL to 690 mL (median [Q1, Q3]: 160 [70.00, 212.50] mL). The correlation analysis revealed positive correlations between the vibration signals and voiding parameters, including the voided volume and RMS (R = 0.768, *p* < 0.001), Qmax and Mmax (R = 0.684, *p* < 0.001), voiding time and signal time (R = 0.838, *p* < 0.001), time to Qmax and time to Mmax (R = 0.477, *p* < 0.001). AI pattern recognition demonstrated high accuracy with all three indicators (precision, recall, and F1 score) surpassing 0.97. **Conclusions**: This AI-assisted vibration-based home uroflowmetry enables accurate voiding parameter measurement and uroflow pattern recognition, showing high precision, recall, and F1-score. It might offer a convenient solution for continuous and subjective bladder monitoring outside clinical settings.

## 1. Introduction

As the population ages, the number of patients reporting lower urinary tract symptoms (LUTS) has steadily increased. For the functional assessment of the lower urinary tract (LUT), bladder diaries (BD) and uroflowmetry (UFM) are widely used non-invasive diagnostic tools.

BD provides data on urinary frequency, bladder capacity, typical voided volumes, urgency and urge incontinence episodes, fluid intake, nocturnal/daytime urine ratio, and bladder pain [1,2]. It evaluates symptoms in both storage and voiding phases. With a balance between adherence and reliability, the optimal duration of 3–7 days BD is recommended [3,4,5]. UFM measures voiding-phase parameters such as the maximum flow rate (Qmax) and voided volume, the uroflow curve, time to Qmax, voiding time, and average flow rate [6]. It is a quite comprehensive and objective evaluation for the voiding phase. Therefore, BD and UFM are included in the guidelines of both the American Urological Association (AUA) and European Association of Urology (EAU) for LUTS evaluation [3,4,6,7].

Although BD and UFM provide key parameters for LUTS evaluation, they have limitations in fully capturing patients’ voiding patterns. BD relies on self-reporting, affecting objectivity and adherence, while office UFM is limited by hospital-based settings and low measurement frequency. Home devices using weighing, fluid-height, or sound-based sensors exist, but face issues like installation, cleaning, and environmental interference. Therefore, we designed a new uroflowmeter based on vibration signals in order to avoid the drawbacks of other technologies, such as less vulnerability to environmental interference, and no need for every-use setup and cleaning.

Thanks to the progress in computing capability and data storage, artificial intelligence (AI) has made exceptional contributions not only in information technology but also in mobile and ubiquitous healthcare. For instance, in medical imaging, deep learning models (a kind of advanced AI model) have been developed for predicting bladder cancer or other urinary tract tumors [8,9]. Relevant studies have demonstrated that these AI models performed excellently in pattern recognition [10]. The application of AI models can assist complex and robust diagnosing processes and even treatment decisions [11]. An AI model (convolutional neural network—CNN) would be used to recognize different patterns of uroflow curves in this study.

To establish a home device for assisting with the diagnosis of voiding dysfunctions, the aims of this paper include the following: (1) to develop an innovative and contact-free technology utilizing vibration signals and artificial intelligence (AI) that provides uroflow curve recognition and voiding parameter measurement; (2) to review the current trend of using of BD and UFM at home; and (3) to compare vibration-based UFM with other technologies.

## 2. Materials and Methods

The research protocol was approved by the institutional review board (IRB: 107-B-10-01). Seventy-six male subjects aged above 20 with informed consent received uroflowmetry (MicroFLO model 2001, LIFE-TECH, Inc., Stafford, TX, USA) for their LUTS. The exclusion criteria included small bladder capacity (<50 mL), external genital malformations, and inability to complete uroflowmetry.

**Figure 1:**
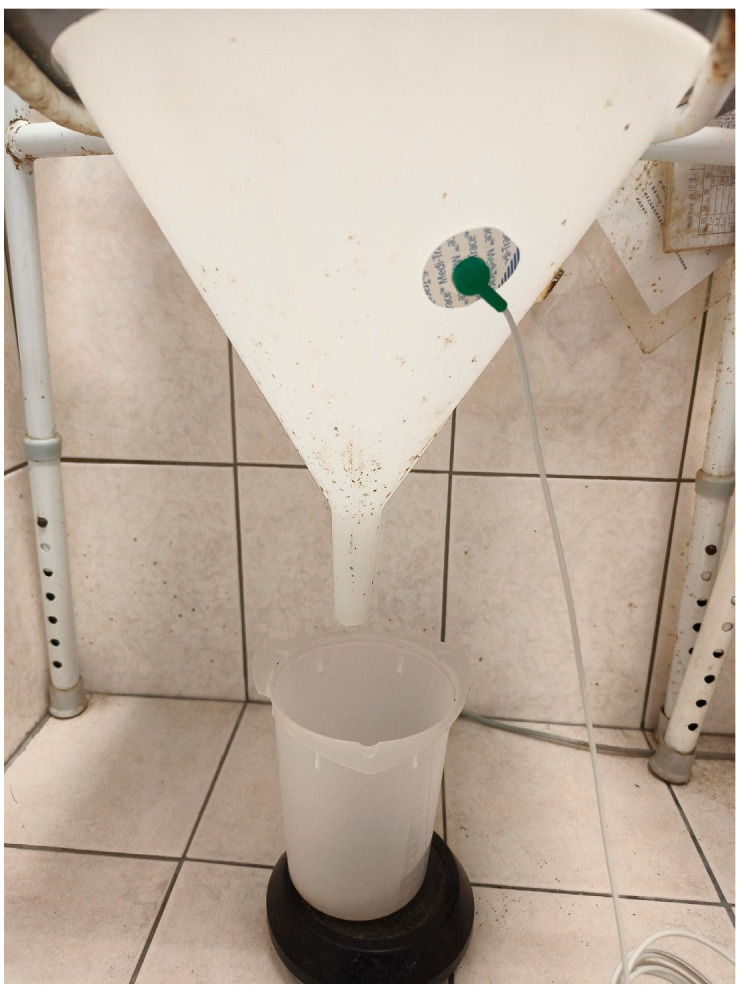
Vibration sensor on the bucket of an office UFM (real-world setting).

A vibration sensor (Kistler triaxial accelerometer 8688A50, Winterthur, Switzerland) is attached to the urine bucket of the office uroflowmeter (Figure 1 real-world picture of the device). When the subject urinates, the accelerometer detects vibration signals and the uroflowmeter measures voiding parameters and depicts the uroflow curve simultaneously. The vibration signals were transferred into a signal processing system [12] and were extracted to be presented as root mean square (RMS) [13]. RMS was used to assess the vibration level in the data preprocessing and to demonstrate RMS magnitude, the maximal amplitude (Mmax), signal time and time to Mmax. Vibration signals during urination were also converted into the mel-frequency cepstrum coefficient (MFCC) [12]. The MFCC spectra display the energy distribution of the physiological vibration signal in the time–frequency domain. This conversion makes the characteristics of various physiological vibration signals more prominent and obvious to be directly identified by human eyes.

The classification model for voiding dysfunction patterns was developed by dividing the database into training and testing datasets. The data is typically split into training and testing sets using K-Fold cross-validation, where the dataset is divided into k equal-sized subsamples. Thus, while the same dataset is reused for training and validation, the cross-validation framework prevents overfitting by systematically validating the model on multiple partitions of the data. In a previous study [12], k = 5 was adopted, with the dataset shuffled prior to splitting. A total of five models were trained and validated, and the averaged validation results were used to assess the model’s predictive reliability. This approach ensures a comprehensive evaluation of the model’s performance across diverse data subsets.

Additionally, an AI model (CNN) combined with uniform manifold approximation and projection (UMAP) dimensionality reduction was used to analyze MFCC spectrums and recognize six patterns of uroflow curves to assist in diagnosing voiding dysfunction. The CNN architecture consists of a series of convolutional layers, fully connected layers, and normalization/dropout mechanisms. The UMAP (uniform manifold approximation and projection) method was employed to project high-dimensional spectrum features onto a 2D manifold, with the dimensionality reduced in this case. Data labels were visualized using color coding to facilitate performance evaluation, as illustrated in the prior literature [12].

Since the complex details of signal processing and AI model application are beyond the scope of this clinical article, they will not be described further. And there are detailed descriptions of signal processing and AI model application in the prior literature [12].

**Figure 2:**
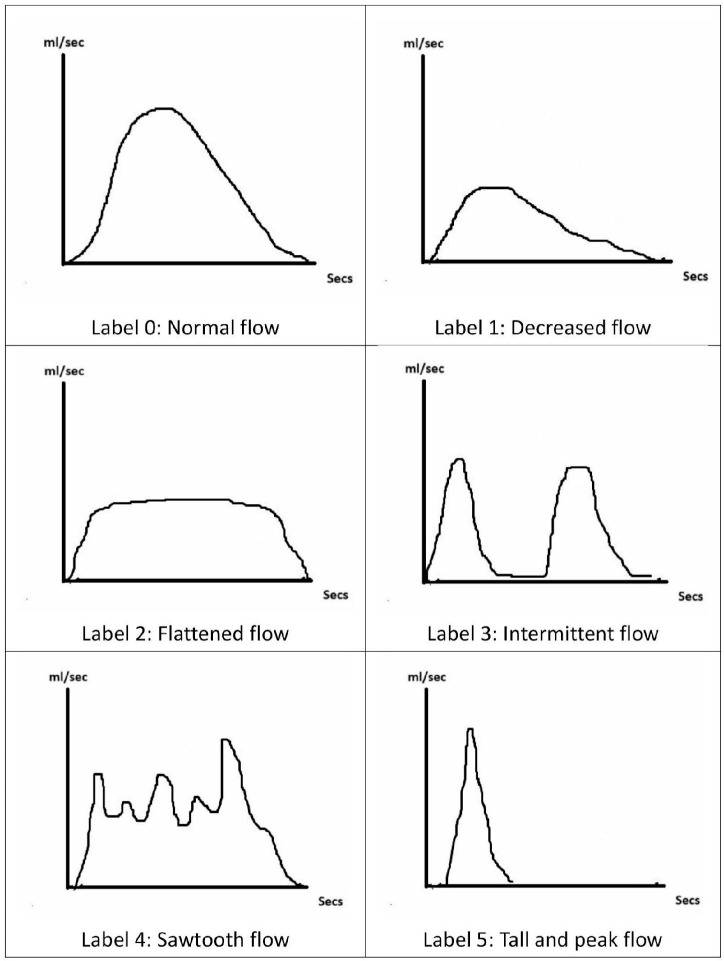
The six common uroflow curve patterns are labeled and classified as follows: 0—normal flow; 1—decreased flow; 2—flattened flow; 3—intermittent flow; 4—sawtooth flow; 5—tall and peak flow. The pattern description is in the text.

As shown in Figure 2, the six common uroflow curve patterns are labeled and classified as:

0 (normal flow, which is the bell-shaped pattern with Qmax ≥ 15 mL/s);

1 (decreased flow, i.e., a prolonged urine voiding time with a low peak flow rate presented);

2 (flattened flow, i.e., the urine flow rate is low and constant until the end);

3 (intermittent flow, i.e., the urine flow rate drops to zero and rise again later);

4 (saw-tooth flow, i.e., the urine flow rate fluctuates greatly but does not drop to zero);

5 (tall and peak flow, i.e., the urine flow rate reaches a high flow peak quickly and declines soon after).

In this study, the physician (YHP) visually determined the uroflow curve patterns based on the raw uroflow curves depicted by UFM. Then the recognition results of the AI model and the physician were compared to make three statistical indicators of accuracy: precision, recall and F1-score. The interpretation of uroflow patterns (by YHP) and the AI trainer (by YTT) was conducted by different researchers, and the process was blinded.

Pearson’s correlation was utilized for correlation analysis. A *p*-value < 0.05 was considered significant.

## 3. Results

Seventy-six subjects (average age: 51.01 ± 14.54 years, BMI: 25.24 ± 3.67 kg/m^2^) were included. The voided volume ranged from 50 mL to 690 mL (median [Q1, Q3]: 160 [70.00, 212.50] mL). The subjects’ Qmax ranged from 4 mL/s to 50 mL/s (average: 16.22 ± 10.68 mL/s). The voiding time ranged from 4 s to 62 s (average: 21.91 ± 12.98 s). The time to Qmax ranged from 0.7 s to 19.5 s (average: 6.26 ± 5.68 s). These four parameters are distributed in a large range (Table 1).

**Figure 3:**
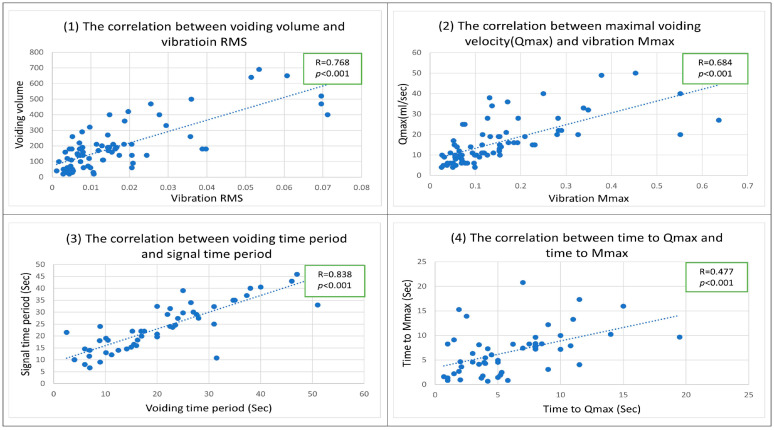
The correlations between the vibration signals and voiding parameters.

The correlation analysis revealed positive correlations between the vibration signals and voiding parameters, including voided volume and RMS (R = 0.768, *p* < 0.001), Qmax and Mmax (R = 0.684, *p* < 0.001), voiding time and signal time (R = 0.838, *p* < 0.001), time to Qmax and time to Mmax (R = 0.477, *p* < 0.001) (Figure 3). That means vibration signals can approximate key voiding metrics of an office uroflowmeter.

Regarding uroflow curve patterns, there were 18, 38, 4, 5, 9, and 2 subjects in pre-classified labels 0, 1, 2, 3, 4, and 5, respectively, as determined by the physician (Table 1).

When compared with the uroflow curve patterns determined by the physician, the recognition results of the AI model (CNN) demonstrated excellent performance with accuracy indicators: precision = 98.26%, recall = 98.19% and F1 score = 98.19%.

## 4. Discussion

After reviewing the prior literature, this study is a novel study using vibration signals impacted by urine stream on the bucket of an office uroflowmeter to measure the voided volume, Qmax, voiding time, time to Qmax, and to depict a uroflow curve [14]. Blaivas et al. previously attached a small-sized vibration sensor on the dorsum of the penis to transform vibration signals into uroflow during urination [15]. This device helps to monitor patients’ voiding status at home. However, this vibration sensor is not contact-free, and contact with the penis may interfere with subjects’ voiding behavior.

The correlation analysis of our study revealed positive correlations (R = 0.768, *p* < 0.001) between the voided volume and RMS, Qmax and Mmax (R = 0.684, *p* < 0.001), voiding time and signal time (R = 0.838, *p* < 0.001), and time to Qmax and time to Mmax (R = 0.477, *p* < 0.001). Compared with Schultz et al.’s results of sound-based UFM, the correlation coefficients of this study are less than theirs (R = 0.91) [16]. It may be the reason that they used an average of 10 sound-based UFM measurements to compare against two office UFM tests. On the contrary, we compared one vibration-based UFM measurement to the corresponding office UFM test in our study. However, our results’ positive correlation and significant *p*-value mean that vibration signals can approximate the key voiding metrics of an office uroflowmeter.

### 4.1. Limitations

**Figure 4:**
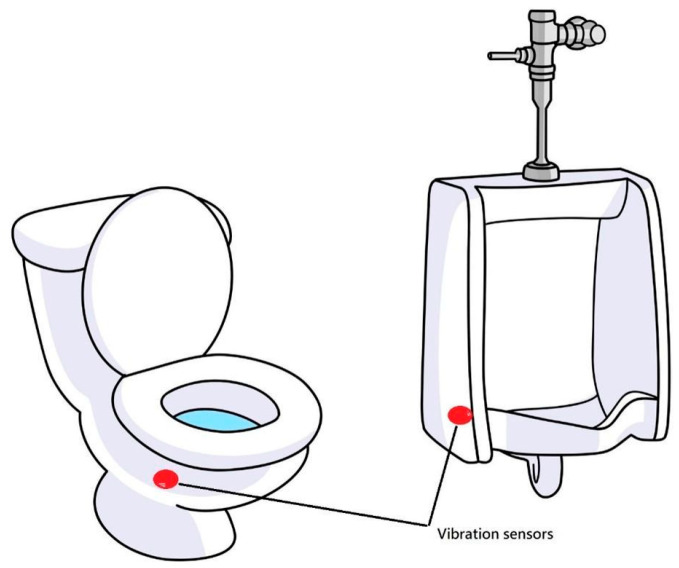
Wireless vibration sensors on a toilet bowl and a urinal.

Even so, there are still some limitations of this study, including only male subjects, a small sample size, and a feasibility test only. The reason for only including male subjects is that a male subject can make the maximal signals corresponding to his voiding [17]. It is important to obtain a high signal/noise ratio for signal processing at this developmental stage of testing feasibility. In a future study, both female and male subjects will be included. After this feasibility test of the vibration-based uroflowmeter, a prototype to fulfill being contact-free will be implemented. To achieve the goal of ubiquitous healthcare, there will be a wireless vibration sensor attached to a toilet bowl or a urinal to sense and transmit vibration signals for the following processing and application (Figure 4). The AI model will be trained and calibrated when collecting vibration signals in different media, e.g., water, a uroflowmetry plastic bucket, a toilet bowl, and a urinal. Then we may be able to develop a correlation coefficient database based on the vibration signals of different media analyses for more precise measurements.

Secondly, some voiding patterns are indeed rare in clinical practice, which is why the number of some specific patterns is relatively low, such as Labels 2–5. As to the training set for the model [12], datasets from major categories (Labels 0 and 1) tend to acquire more discriminative features and information compared to minor categories (Labels 2–5). This imbalance may compromise the model’s ability to accurately identify underrepresented classes, even if the overall accuracy appears high. To address this class imbalance, naive random oversampling was employed. This technique involves randomly selecting examples from minority classes (Labels 2, 3, and 5) with replacement and incorporating them into the training dataset, while ensuring that the testing dataset (unseen data) remains untouched. By generating additional synthetic samples for underrepresented classes, the model’s generalization capacity was enhanced while mitigating overfitting risks. However, based on the model, the precision, recall, and F1-score all showed strong performance, indicating that this AI model can still provide a certain level of clinical support.

Moreover, the interpretation of uroflow patterns (by YHP) and the AI trainer (by YTT) was conducted by different researchers, and the process was blinded. However, there is no inter-rater variability at this stage. We will conduct an inter-rater variability assessment in a further study.

### 4.2. AI Applications in Home UFM and Urology

Regarding uroflow curve pattern recognition, the AI model (CNN) demonstrated excellent performance and accuracy compared to the patterns identified by physicians. Prior home UFM applications included AI models, such as the Proud P^®^ using prediction algorithms for voiding parameters [17], and Jin J et al. applying long short-term memory (LSTM) to compare office uroflowmetry data with voiding sounds [18]. Many home UFMs lack the pattern recognition of the uroflow curve, requiring a visual expert assessment [16,17]. Inter- and intra-rater reliability in curve pattern recognition remains a research concern [19]. AI may advance this area, particularly through big data from repeated home UFM measurements.

AI has been widely applied in urology, including in the diagnosis of benign prostatic hyperplasia, urothelial and prostate cancers [10], machine learning for bladder diseases [11], and models supporting female urinary incontinence management [20]. It serves as an assistive tool for diagnosis and care across top-tier institutions and rural settings. Given limitations in manpower, time, and funding, AI is a valuable supplement to conventional methods.

### 4.3. Big Data and Repeated Measurements in Home UFM

As we know, big data is essential for AI model training and application. Furthermore, repeated measurements of home UFM generate big data. It was even discussed that a large amount of at-home data is more reliable than one-time in-office tests [16]. Actually, we have seen many presentations about home UFM in the latest AUA annual meetings and literature [21,22,23,24]. Also, several commercial home UFMs were demonstrated in the 2024 AUA industry exhibition hall, providing repeated measurements of voiding parameters and uroflow curves [16,17,19,21,22,23,24]. And this way of repeated measurement can draw a clearer picture of our patients’ daily-life voiding, but without the anxiety and stress from office UFM tests [24].

Indeed, there is a need for home uroflowmetry to fulfill repeated measurements for the convenience of solving several clinical and practical headaches for the practicing urologist [16]. Since there are several types of at-home UFM emerging in the market, what are the differences between the different technologies?

### 4.4. Home UFM of Different Technologies

All kinds of home UFM with different technologies have common advantages: automatic recording, repeated measurement, and big data. Nevertheless, each different technology has different features and limitations when implemented for home UFM. Table 2 shows some features of the different technologies, including accuracy, vulnerability to surrounding interferences, uroflow curve pattern recognition, AI algorithms, and contact-free. In terms of accuracy, all technologies have demonstrated comparable results with office UFM and even obtained FDA approval. Due to the inborn weakness, sound-based technologies are vulnerable to surrounding interferences, especially noises and barriers. Also, the urine stream must be voided into a water-filled commode, not a urinal, for the app to record sound [16]. And the sound of voiding differs between men and women, likely due to differences in the anatomy and posture during urination. Males could make louder sounds during urination due to the standing position, which has no barrier for decreasing sound. However, different models have been developed for both sexes and applied accordingly to solve these problems [17]. On the contrary, vibration-based technology may avoid the barrier to diminish sound transmission. And vibration transmission should be more direct than sound transmission. Sound is transmitted through the air, while vibration is transmitted by the toilet bowl or urinal to the sensor. Sound-based technologies are more vulnerable to the surrounding noise than vibration-based [21]. After reviewing the prior literature, only sound- and vibration-based technologies have applied AI models or algorithms for uroflow curve pattern recognition [12,17,18]. For the user’s intuitive and contact-free measurements, sound- and vibration-based technologies have these advantages because they do not need installation and cleaning in each measurement.

### 4.5. An Ideal Model for Home UFM

In the future, a vibration-based UFM could be applied for remote monitoring and diagnostic support. An ideal model combining UFM and BD functions may integrate water intake logs and urgency records. As Lai et al. proposed UFM as a first-line test for nephropathic patients due to its simplicity, repeatability, non-invasiveness, and low cost, our model could serve as an effective implementation for this purpose [25].

**Table 2:** The comparison of different technologies for home UFM.

Features\Technologies	Weighing (Gravimetric)	Height Sensor Stream Dx	Sound	Vibration
Accuracy	FDA approved [22]	Non inferior to existing methods [26]	Correlation with office UFM (R = 0.91) [16]; Prediction rate of 99% [18]	Uroflow curve pattern recognition accuracy > 0.98 [12]
Vulnerability to the surrounding interferences	X	X	V [17]	X
Uroflow curve pattern recognition	X	X	V [18]	V
AI algorithm/model	X	X	V [17,18]	V
Contact-free (no need for installation/cleaning)	X	X	V	V

X: unavailable, V: available.

## 5. Conclusions

This vibration-based UFM measures voiding parameters with high correlation to traditional methods. And the AI-assisted uroflow pattern recognition demonstrates high precision, recall, and F1-score. It might provide a convenient solution for continuous and subjective voiding monitoring outside clinical settings, offering a valuable reference for physicians.

## Figures and Tables

**Table 1:** The distribution of the subjects in six uroflow curves.

**Patients (*n* = 76)**	**Data**
Age (years)	51.01 ± 14.54
BMI (kg/m^2^)	25.24 ± 3.67
Median voided volume (mL)	160 [70.00,212.50]
Average Qmax (mL/s)	16.22 ± 10.68
Average voiding time (s)	21.91 ± 12.98
Average time to Qmax (s)	6.26 ± 5.68
**Uroflow patterns**	**Numbers of patients**
Normal (label 0)	18
Decreased flow (label 1)	38
Flattened flow (label 2)	4
Intermittent flow (label 3)	5
Sawtooth flow (label 4)	9
Tall and peak flow (label 5)	2

## Data Availability

Research data supporting the reported results are available from the corresponding authors upon reasonable request.

## Abbreviations

The following abbreviations are used in this manuscript:

AI	artificial intelligence
ANN	general neural network
AUA	American Urological Association
BD	bladder diary
BMI	body mass index
CNN	convolution neural network
EAU	European Association of Urology
LSTM	long short-term memory
LUT	lower urinary tract
LUTS	lower urinary tract symptoms
MFCC	mel-frequency cepstrum coefficient
Mmax	maximal amplitude
Qmax	maximal flow rate
RMS	root mean square
UFM	uroflowmetry
UMAP	uniform manifold approximation and projection
